# Malaria elimination in Haiti by the year 2020: an achievable goal?

**DOI:** 10.1186/s12936-015-0753-9

**Published:** 2015-06-05

**Authors:** Paul Jacques Boncy, Paul Adrien, Jean Frantz Lemoine, Alexandre Existe, Patricia Jean Henry, Christian Raccurt, Philippe Brasseur, Natael Fenelon, John B Dame, Bernard A Okech, Linda Kaljee, Dwayne Baxa, Eric Prieur, Maha A El Badry, Massimiliano S Tagliamonte, Connie J Mulligan, Tamar E Carter, V Madsen Beau de Rochars, Chelsea Lutz, Dana M Parke, Marcus J Zervos

**Affiliations:** Laboratoire National de Santé Publique, Rue Chardonnier #2 and Delmas 33, Port-au-Prince, Haiti; Direction d’Épidémiologie, de Laboratoire et de Recherches, Port-au-Prince, Haiti; Programme National de Contrôle de la Malaria, Port-au-Prince, Haiti; Point focal OHMaSS/Programme National de Contrôle de la Malaria, Port-au-Prince, Haiti; Institute of Research for Development, Dakar, Senegal; University of Florida, Gainesville, FL 32611 USA; Henry Ford Health System, 2799 W. Grand Blvd, Detroit, MI 48202 USA; Wayne State University, Detroit, MI 48201 USA; Oakland University William Beaumont School of Medicine, Rocheste, MI 48309 USA; Laboratoire Vac4All, Hôpital Cochin, 75014 Paris, France

**Keywords:** Malaria, Elimination, Haiti

## Abstract

Haiti and the Dominican Republic, which share the island of Hispaniola, are the last locations in the Caribbean where malaria still persists. Malaria is an important public health concern in Haiti with 17,094 reported cases in 2014. Further, on January 12, 2010, a record earthquake devastated densely populated areas in Haiti including many healthcare and laboratory facilities. Weakened infrastructure provided fertile reservoirs for uncontrolled transmission of infectious pathogens. This situation results in unique challenges for malaria epidemiology and elimination efforts. To help Haiti achieve its malaria elimination goals by year 2020, the Laboratoire National de Santé Publique and Henry Ford Health System, in close collaboration with the Direction d’Épidémiologie, de Laboratoire et de Recherches and the Programme National de Contrôle de la Malaria, hosted a scientific meeting on “Elimination Strategies for Malaria in Haiti” on January 29-30, 2015 at the National Laboratory in Port-au-Prince, Haiti. The meeting brought together laboratory personnel, researchers, clinicians, academics, public health professionals, and other stakeholders to discuss main stakes and perspectives on malaria elimination. Several themes and recommendations emerged during discussions at this meeting. First, more information and research on malaria transmission in Haiti are needed including information from active surveillance of cases and vectors. Second, many healthcare personnel need additional training and critical resources on how to properly identify malaria cases so as to improve accurate and timely case reporting. Third, it is necessary to continue studies genotyping strains of *Plasmodium falciparum* in different sites with active transmission to evaluate for drug resistance and impacts on health. Fourth, elimination strategies outlined in this report will continue to incorporate use of primaquine in addition to chloroquine and active surveillance of cases. Elimination of malaria in Haiti will require collaborative multidisciplinary approaches, sound strategic planning, and strong ownership of strategies by the Haiti Ministère de la Santé Publique et de la Population.

## Background

Haiti and the Dominican Republic, which share the island of Hispaniola, are the last locations in the Caribbean where malaria still persists [1]. Malaria is an important public health concern in Haiti with 17,094 reported cases in 2014 by the Service de Suivi et d’Evaluation of the Programme National de Contrôle de la Malaria (PNCM) (private database); however, it has been estimated that up to 220,000 cases may be present, with an estimated 80 % of the population at risk of acquiring an infection [2–8]. In patients presenting to clinics in Haiti with undifferentiated acute febrile illness, 3 to 47 % were malaria positive [3–12]. The morbidity and economic impact of malaria is enormous, with infections being both a cause and a result of poverty [13].

In Haiti, malaria infections have been found to be predominantly caused by *Plasmodium falciparum* [1], but *Plasmodium malariae* appears sporadically [14]. *Anopheles albimanus* is the confirmed vector [15], but *Anopheles pseudopunctipennis* may also be a potential vector [16]. Studies in Haiti on the susceptibility of *P. falciparum* to chloroquine date back thirty years [17] and these studies continue to the present day. This anti-malarial is considered to the present day by the Ministère de la Santé Publique et de la Population (MSPP—Haitian Ministry of Public Health and Population) as the first-line drug of choice for treating uncomplicated malaria. Further, on January 12, 2010, a record earthquake devastated densely populated areas in Haiti including many healthcare and laboratory facilities. International aid has supported reconstruction efforts yet, even now, large densely populated settlements and weakened infrastructure provides fertile reservoirs for uncontrolled transmission of infectious pathogens. This situation resulted in unique challenges for malaria elimination efforts. Air travel between Haiti and other countries from international aid agencies, while welcomed, actually offered the potential for rapid dissemination of novel or drug-resistant malaria. Knowledge of the origin of novel strains is needed to detect the potential importation of drug resistant strains.

Over the past decade there has been renewed interest in eliminating malaria from Hispaniola, with a bi-national agreement recently adopted between the Dominican Republic and Haiti to eliminate malaria by 2020 [2]. Reported cases of malaria in the Dominican Republic have reached a 15-year low of 952 cases in 2012; however, 1/3 of these cases were thought to be imported directly from Haiti [2]. Thus, control of malaria in Haiti will be key to sustainable malaria elimination for the entire island. However, current programmes to eliminate malaria in Haiti are limited by lack of active surveillance of infections.

To help Haiti achieve its malaria elimination goals by year 2020, the Laboratoire National de Santé Publique (LNSP) and Henry Ford Health System (HFHS), in close collaboration with the Direction d’Epidemiologie, de Laboratoire et de Recherches (DELR) and the Programme National de Contrôle de la Malaria, hosted a scientific meeting on “Elimination Strategies for Malaria in Haiti” on January 29-30, 2015 at the National Laboratory in Port-au-Prince, Haiti. The meeting was followed by the Forum Scientifique, hosted by the DELR, which highlighted presentations on malaria research in Haiti. The meeting brought together laboratory personnel, researchers, clinicians, academics, public health professionals, and other stakeholders to discuss main stakes and perspectives on malaria elimination. The two-day meeting included presentations by representatives from the MSPP, PNCM, DELR, LNSP, HFHS, University of Florida, and Centers for Disease Control and Prevention (CDC).

### Situational review: malaria on the island of Hispaniola: current surveillance efforts

Eradication efforts for malaria on the island of Hispaniola were almost completely successful in the 1960s. The Dominican Republic reduced incidence of malaria to 21 cases in 1968, whereas Haiti reduced positive rates from blood slides to below 1 % despite screening of well over one million people that same year of a total population of five million inhabitants. However, the island of Hispaniola reported 33,664 confirmed cases of malaria by *P. falciparum* in 2011, of which 4.8 % were in the Dominican Republic and 95.2 % in Haiti (Henry PJ, personal communication). Although malaria is considered as one of the 10 principal causes of death in Haiti, there were only 10 deaths caused by malaria in the Dominican Republic in 2010.

Both countries engage in current interventions. In the Dominican Republic, the programme consists of vector control for prevention, with diagnosis and treatment of active cases. The Dominican Republic’s current interventions have strengths and weaknesses. Strengths include testing all suspected cases, communicating each case to the central level, enhanced surveillance for cases in high-risk regions and among very exposed populations, directly observed treatment, targeted activities for vector control, and a high level of comprehension of the malaria risk by the population resulting in active participation in interventions. Weaknesses include limited funding for low- to medium-risk zones, the passive surveillance system is not yet automated, and the utilization of microscopes to diagnose cases.

In Haiti, current interventions include measures of vector control, including notably the distribution of insecticide-treated nets (ITNs) with the support of The Global Fund, as well as partners including local NGOs and the CDC, that also cooperate with the government to improve surveillance systems and case management. Strengths of the current interventions in Haiti include: the recent adoption of rapid diagnostic tests (RDTs) and the treatment combination of chloroquine plus primaquine, as well as entomological studies and larvicide operations conducted as needed, with the involvement of international partners. Weaknesses include the lack of training and dissemination of the most recent norms and the national strategic plan, the communication of suspected cases rather than confirmed cases, and the absence of a mechanism to transfer information on individual cases to the central level and lack of related feed-back from central to local settings. Further, the absence of active case detection and absence of dissemination or insufficient information prevents the targeting of key interventions.

Opportunities exist for interventions in both countries. For example, the growing political interest and engagement for bi-national collaboration, as well as a new CDC project at the Haiti— Dominican Republic border to use a community-testing model, show great prospects. However, challenges remain, including instability and migration that are caused by natural disasters, and the lack of funding.

### Malaria in Haiti in 2014

Traditionally, microscopy was the only technique recognized by the Haitian Ministère de la Santé Publique et de la Population for malaria detection. However, after the January 2010 earthquake, international actors brought RDTs, and a 2010 survey compared the performance of several RDTs to microscopy and found good results for certain brands. Thus, three brands of RDTs were authorized by the Ministry of Health on April 12, 2010: First Response, CareStart, and SD Bioline. Currently for year 2015, three techniques are used in Haiti for confirmed diagnosis of malaria: 1) microscopy, which serves as the reference test; 2) RDT; and, 3) polymerase chain reaction-based assays (PCR). However, PCR is used only by the National Laboratory in the evaluation of certain studies; it is not used for diagnostic purposes.

In 2014, there were 17,094 reported cases of malaria in Haiti. Of these, 10,599 or 62 % were identified by microscopy, whereas 6,495 (38 %) cases were diagnosed by RDT. The number of cases may be lower as a result of limitations of testing and issues with case reporting. The lower number of cases identified by RDT is attributed to the fact that many institutions in Haiti lack access to the tests. It is also suspected that there is a percentage of false positives reported via microscopy, since uninfected red blood cells may appear to contain a parasite where staining is poor or the observer is less experienced.

**Fig. 1:**
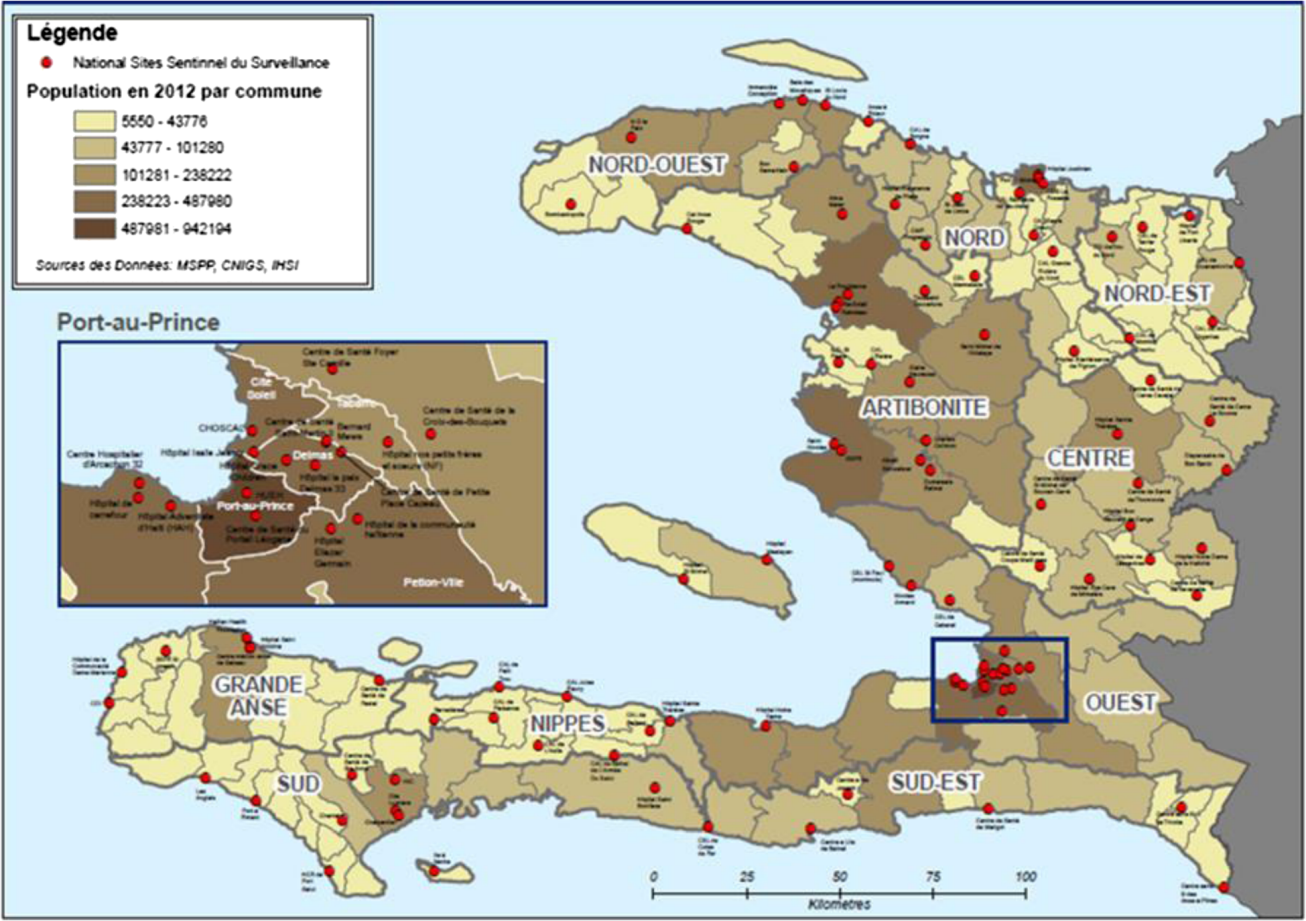
National Sentinel Surveillance Sites in Haiti

Table 1 displays the distribution of cases of malaria in Haiti in 2014 by geographic department; Fig. 1 maps the national sentinel surveillance sites in Haiti. Of these, the villages with the highest number of cases include: Croix des Bouquets [Ouest—1,941], Delmas [Ouest—1,481], Petit-Goave [Ouest—972], Port-au-Prince [Ouest—763], Dame-Marie [Grande-Anse—641], Gros-Morne [Artibonite—470], Port de Paix [Nord-Ouest—393] and Jeremie [Grande-Anse—367]. These statistics represent the hospitals or clinics where cases of malaria are confirmed and reported to the MSPP.

**Table 1:** Number of Cases of Malaria in Haiti in 2014 by Geographic Department

Department	Number of cases	Percentage
Ouest	8,406	49 %
Grande’Anse	2,143	13 %
Artibonite	1,944	11 %
Sud	1,367	8 %
Nord-Ouest	940	5 %
Nord	936	5 %
Centre	535	3 %
Nord-Est	297	2 %
Sud-Est	367	2 %
Nippes	159	1 %
TOTAL	17,094

However, there is a flaw in this representation: often, people come from very far away to receive quality care at larger hospitals. Thus, these data may be misleading for elimination strategies, as they do not accurately represent the locations where the malaria transmission occurred. For example, the city of Kenscoff in the Ouest Department reported 25 cases; however, the climate at this elevation is not consistent with parasite development and transmission by the Anopheles mosquito (the malaria vector); thus, these patients are most likely contracting malaria in other areas at a lower altitude. Updated data and field interventions to determine where the vector resides are essential to target elimination strategies for malaria in Haiti.

### Laboratory diagnosis of malaria

Key to achieving malaria elimination is the proper laboratory diagnosis of malaria. An ideal test provides easily interpreted results, quickly, accurately, specifically and with great sensitivity. Specificity and sensitivity are of greater importance if the goal of eradication is to be achieved. The first step of assay selection is to recognize advantages and limitations of tests conducted. Malaria microscopy, rapid diagnostic testing and several molecular methods, that have utility in resource-limited settings, were reviewed.

The gold standard for malaria diagnosis is microscopic detection of *Plasmodium* morphologies [18]. Skilled microscopists can readily identify infection and differentiate species at concentrations below 100 parasites per microlitre of blood utilizing both thick and thin blood smear modalities. Microscopy lends itself as an efficient tool for calculating parasite densities and determining the efficacy of therapeutic interventions. One challenge for microscopy is that as the incidence of malaria cases decreases, the labor force for detection becomes less practiced in the ability to detect rare cases. This can be addressed via a quality assurance programme that includes a centralized proficiency-testing component; however, equipment and skilled labour force requirements make microscopy less viable as the sole diagnostic tool.

The availability of a range of rapid diagnostic tests offers an attractive alternative to microscopy for the detection of malaria parasite antigens and enzymes [19]. As a field diagnostic tool they have several advantages in that they provide rapid results, can be administered outside of the health care setting, do not require an expensive equipment infrastructure and are simple to perform and interpret. Disadvantages include vulnerability to temperature and humidity, varying sensitivities and specificities, difficulty identifying a new infection from an effectively treated infection and inability to calculate parasite density. For these reasons, RDT may serve a greater purpose by being a component in a screening and confirmatory testing algorithm in concert with microscopy, particularly in eradication scenarios.

There are a variety of other molecular testing methods for malaria that in general increase the sensitivity and specificity of detection. However, most require specialized equipment, reference laboratory availability, and are technically demanding with unsatisfactory turn-around-times. Several recently reported techniques with more satisfactory characteristics for malaria testing are PET-PCR [20, 21], RNA Hybridization Assay [22], Ligase Detection Reaction-Fluorescent Microsphere (LDR-FM) [23], Loop Mediated Isothermal Amplification (LAMP) [24] and a malaria biosensor [25]. Reported sensitivities and specificities are up to 100 % with some having more advantageous turn-around-times. In particular, the LDR-FM assay has the advantage of detecting single nucleotide polymorphisms thereby identifying chloroquine and amodiaquine resistant organisms. The LAMP assay, in comparison, has several beneficial characteristics. LAMP is the isothermal amplification of target nucleic acid utilizing looping primer oligonucleotides. The incorporation of dyes such as SybrGreen or hydoxynaphthol blue allow for colorimetric detection of amplified product within the reaction tube. The sensitivity and specificity of this technique is reported to be 100 %. This tool is of value in that it reduces PCR contamination, does not require elaborate equipment other than an isothermal heating unit and has a relatively rapid turn-around-time. The primary disadvantage is the requirement for electrical power.

Malaria testing should be cost effective, sensitive, specific, have utility for therapeutic monitoring, and yield similar results whether conducted in the field or reference laboratory. All testing can be optimized by implementation of quality assurance programmes, encompassing pre-analytic, analytic and post-analytic phases of testing, and application of testing algorithms for screening and confirmatory testing.

### Entomological capacity building for the surveillance of mosquito vectors

There is an urgent need to increase the entomological research capacity that would enable detailed bioecological studies on mosquitoes in Haiti to aid in malaria control efforts. A search of PubMed revealed very few published studies on malaria mosquitoes in Haiti, indicating a lack of research activities related to this topic. In the available literature (published and grey literature), only six mosquito species are mentioned: *An. albimanus, An. pseudopunctipennis, Anopheles vestitipennis, Anopheles grabhami, Anopheles crucians*, and *Anopheles argyritarsis* (Lutz C and Okech B, personal communication). The major malaria vector in Haiti is *An. albimanus*, which has been well documented to have a bite preference for animals over humans. There is a need to understand in greater detail all other potential malaria vectors in Haiti as serious discussions commence on malaria elimination strategies. Entomological studies on vector-borne diseases in Haiti are hampered by the severely limited infrastructural capacity, the absence of highly trained entomologists at the masters and doctorate level and, finally, the limited funding from local and outside agencies.

The University of Florida vector surveillance programme activities aim to strengthen the malaria surveillance capability by enhancing the entomology laboratory capacity in Haiti. The entomological research capacity in Haiti was launched by University of Florida following a model from Africa where the President’s Malaria Initiative has invested substantially in entomology infrastructure by converting shipping containers into movable insectarium or “insectary in a box”. The “insectary in a box” facilities were needed to monitor the insecticide susceptibility of malaria vectors in Africa. Following this model, with collaboration and support from the LNSP, the CDC, and the Armed Forces Health Surveillance Centers Global Emerging Infections Surveillance, the University of Florida oversaw the building of such an “insectary in a box” within the compound of the LNSP with a completion date of September 29, 2014. This compound also houses the Department of Epidemiology and Laboratory Research. The insectary has three rooms: a mosquito species identification room, an adult mosquito holding chamber with air conditioning and humidifier, and the larval mosquito room rearing mosquitoes. Currently the insectary has a colony of *An. albimanus* mosquitoes that are being maintained by two well-trained technicians working in the insectary.

The laboratory is a critical resource for elimination of malaria in Haiti by serving to support surveillance, research, and intervention work conducted in concert with the MSPP, University of Florida scientists, U.S. Department of Defense, and other collaborating investigators in studying and preventing insect borne diseases in Haiti. Since the building of the insectary, University of Florida and CDC researchers have collaborated with the PNCM in Haiti to provide training for field mosquito workers in Haiti. Each of Haiti’s 10 departments has mosquito field workers who report directly to the PNCM. With a developed training plan following World Health Organization training manuals, these field mosquito workers were trained on sampling strategies, basic mosquito identification and storage, and mosquito rearing.

The next steps for utilizing the insectary include initiating mosquito surveillance in concert with Haitian government priorities. This will include insecticide susceptibility studies to support implementation of wide scale vector control operations in Haiti, continued training to increase the number of Haitian mosquito control scientists, and providing support to other collaborators and scientists interested in studying and preventing vector-borne diseases in Haiti.

### Genetic diversity of the malaria parasite in Haiti to aid in elimination efforts

In recent years, certain strains of *P. falciparum* have shown the presence of the gene for resistance to chloroquine in the Artibonite region [6, 26] as well as in travellers returning from Haiti [27]. Furthermore, recent studies have shown the presence of the S108N mutation on some strains of *P. falciparum,* a mutation that confers some degree of resistance to pyrimethamine [28], as well as the presence of a Y184F mutation on the *pfmdr1* gene of *P. falciparum* which has implications on the susceptibility of the parasite to drugs [29]. It is thus necessary to continue studies genotyping strains of *P. falciparum* in different sites with active transmission in Haiti.

Toward the goal of elimination of malaria in Haiti, University of Florida investigators are surveying the population of *P. falciparum* to identify drug resistance loci in the parasite population as a part of a study of the allelic diversity of the parasites and the geographic distribution of that diversity. These studies have employed microsatellite analyses as well as deep sequencing of the parasite genome for expanded single nucleotide polymorphism (SNP) analyses as well as phylogeography. Further, in this work, a PCR-based method for detecting virtually all blood stage infections with *P. falciparum*, even the infections of asymptomatic carriers, was implemented.

Conclusions from results obtained to date are that of 79 isolates analysed for the chloroquine resistance mutation (CQ^R^) K76T, none carried this resistance allele. The geographic distribution of samples tested was heavily centered in Port-au-Prince and vicinity where over half of the samples were obtained with the remaining samples coming from Jacmel, Artibonite, Nippes, and Cap Haitian [29]. With the comparatively small sample size, University of Florida investigators had only ~50 % chance of identifying a sample with a resistance locus, if the true prevalence were 1 %, thus University of Florida investigators are only able to conclude that the prevalence is low which is consistent with reports of chloroquine resistant malaria in travelers following the 2010 earthquake [27]. Similarly a subset of these samples was examined for mutations N86Y, S1034C, N1042D and D1246Y in *pfmdr1*, and none were found. Thus, none of the isolates have a *pfmdr1* allele that would enhance resistance to chloroquine or to mefloquine or amodiaquine [29].

The genetic diversity of 85 Haitian *P. falciparum* isolates, with a geographic distribution of origin as described above, was assayed using 12 microsatellite loci [30], (Carter *et al.*, personal communication). There was a low percentage of multiple infections detected affirming low transmission rates, but a high allelic diversity was detected. The similar distribution of alleles found in isolates from Terre Noire, Leogane, and Jacmel suggests free gene flow between these parasite populations. Efforts are underway to expand these studies to include parasite isolates from more remote regions of Haiti and to expand the examination of allelic diversity to include SNPs identified through deep sequencing of the genome. Sampling has been expanded to include parasites from Grand’ Anse with continuing efforts to obtain isolates from other remote regions. The allelic diversity within this set of samples will be compared with that of the samples previously analysed. Informative SNPs which allow us to discriminate between the *P. falciparum* parasites found on Haiti from those present in other regions of the world are being sought, as well as SNPs that would define isolated subpopulations of parasites present within Haiti should they be present. Genome sequence data are being employed to determine the genetic relationship of *P. falciparum* isolates from Haiti with those from around the world as reported by Manske *et al.* [31]. These data will allow identification of drug resistance mutations and predisposing genetic factors for the development of drug resistance in the parasite population and provide data on the diversity of genes encoding potential vaccine antigens should a malaria vaccine become available.

Initial results, from sequencing the genome (138x coverage) of isolate Leo1 derived from a patient in Leogane, identified more than 20,000 SNPs in coding sequences as compared to the reference clone 3D7. Of these more than 8,000 were homozygous non-synonymous SNPs. Not surprisingly these data, when compared by nearest neighbor or principal component analyses with those of the MalariaGen Project from Africa and Southeast Asia [31], indicated that this isolate was most closely related to isolates characterized by the MalariaGen Project from West Africa. Genetic diversity data including both microsatellite and SNP analyses will inform elimination efforts identifying introduction of parasites from foreign locations and describing the translocation of parasites within Haiti should genetic subpopulations be identified.

Critical to elimination efforts will be the detection of all individuals infected with the parasite, so that they may be treated. Of all malaria infections, the most difficult to identify are those in asymptomatic individuals who have no reason to seek medical care. These are those in the earliest stages of infection and those who have become persistently infected and who no longer have obvious symptoms. A recent review has described the potential for *P. falciparum* to persist in an asymptomatic host for more than a decade [32]. These infections were either discovered as transfusion or transplant-transmitted malaria or with the appearance of symptoms long after leaving a malaria endemic area. Authors of this review drew the conclusion that is of critical importance to elimination efforts in Haiti: *“Even if only a tiny proportion of untreated or partially treated infections become indolent, this represents a huge potential reservoir of sustained transmission”.*

Removing this threat to elimination efforts will require treating literally everyone in the population or detecting and treating those who are asymptomatic carriers. The surprising number of severely anemic individuals among children and pregnant women in the rural population where University of Florida investigators have monitored for malaria infections may present a concern for use of primaquine in a mass drug administration strategy, since up to 20 % of the population is G6PD deficient [33]. Testing will require obtaining a blood sample, allowing assessment of anemia before administering primaquine, but successful elimination via diagnosis and treatment will require an ultrasensitive test that reliably detects virtually all infected individuals. Reliability of detection decreases at lower parasitaemia for virtually all tests, since one or more parasites or the target to be detected must be in the sample to obtain a positive test result. A molecular, PCR-based test for the ribosomal RNA of the parasite promises to be the most sensitive test available for detecting infected individuals. Using rRNA as the target overcomes limitations of genomic DNA targets, since ribosomal RNA molecules are among the most abundant stable nucleic acid molecules in the cell, estimated to be present at ~10,000 copies per parasite [34]. Thus, lysis of a single parasite in the sample releases ~10,000, freely dispersing targets, which allows testing of a small fraction of the nucleic acids extracted from a larger volume of blood, up to 1 ml. Results from the University of Florida investigators’ laboratory indicate the ability to routinely detect a single parasite in 1.0 ml of whole blood using a method very similar to that of Kamau *et al.* [35] (El Badry *et al.*, personal communication).

The results of these studies provide critical data needed to guide and monitor malaria elimination by: 1) determining accurately the point prevalence of both symptomatic and asymptomatic infections with *P. falciparum* in communities across Haiti in times of both peak malaria transmission and minimal malaria transmission; 2) profiling human populations most likely to have asymptomatic infections; 3) monitoring changes in allelic diversity in time and space; 4) identifying isolated parasite populations, if they exist presently or when they arise during elimination; 5) monitoring changes in point prevalence during elimination; and 6) monitoring the appearance and spread of drug resistant haplotypes during elimination should they emerge.

### *Plasmodium falciparum* gametocyte carriers amongst the habitants of five departments of the South of Haiti

A study by Raccurt *et al.* [36] details community surveys for malaria conducted by the LNSP between May 2010 and October 2013. The surveys were conducted in five Departments: Ouest, Sud-Est, Nippes, Grande’ Anse and Sud. Of 5,342 people surveyed, 248 were positive, 170 were carriers of trophozoite forms, and 170 were carriers of gametocytes. When broken down by age, all levels had similar percentages of gametocytes (~3.5 %) except for the group of age 50 or higher, which decreased to 1.9 %. The study also noticed seasonal variations between the rainy seasons (trophozoites: 4.1 % and gametocytes: 2.4 %) and the dry seasons (trophozoites: 9.1 % and gametocytes: 6.3 %).

The population adhered very strongly to the survey (only 0.9 % refused). The study found large variations of indicators by locality: malaria is territorial and seasonal, so periods of transmission vary. Malaria represents about 5 % of febrile cases seen in consultation. The higher prevalence of malaria in all age groups except for those age 50 and up demonstrates that all ages must be studied, not just children.

Several recommendations can be made based on the results of this study. A critique of current official reporting methods is that cases are reported based on the hospital where the diagnosis occurred—not where the patient actually resides (which often is very far away from the hospital). This biases the statistics which consequently impacts where malaria elimination strategies are targeted. Thus a better identification mechanism of active households where transmission occurs is needed. Additionally, contact between humans and vectors and the dynamics of transmission must be studied. The control strategies must be targeted and build on the appropriate results of scientific studies.

### Survey of *Plasmodium falciparum* chloroquine resistant genotypes Pfcrt in 6 foci of transmission in the South of Haiti

This study surveyed six foci of malaria transmission in Haiti in the Sud-Est, Ouest, and Grande’ Anse departments. The finger prick blood specimens of 45 malaria positive patients were transferred on Whatman filter paper on three spots for each clinical sample. Filters were air-dried and stored in individual plastic pouches with a desiccant pack at 4° in the dark. Genomic DNA of *P. falciparum* was extracted from a 50 mm^2^ surface of blood-saturated filter by soaking the paper in 100 μL of Tris-EDTA buffer. Filters were incubated at 50 °C for 20 min followed by a rapid step of mild vortex agitation, heated at 97 °C for 20 min. DNA samples were stored at -20 °C until use.

For the molecular analysis, the polymorphism K76T on the CRT protein was analysed by restriction fragment length polymorphism (RFLP) with *Apo* I enzyme on an amplicon of the *Pfcrt* gene. The parasite genomic DNA extracted from the blood samples was amplified by a nested PCR. The amplifications were digested with *Apo* I to verify the absence of substitution of the lysine residue at position 76 by a threonine giving two bands of 124 and 68 base pairs long on a 3 % agarose gel electrophoresis. Two clinical samples from Senegal with a known CQ-sensitive and CQ-resistant phenotype were used as controls.

The 45 positive samples analysed were amplified by nPCR and analyzed by RFLP. The results showed that all were digested with *Apo* I whereas, as expected the Senegalese CQ-resistant control was not. The detection of *pfcrt*, K76 T mutation responsible of resistance to chloroquine was negative for all samples tested.

In conclusion, the molecular analysis confirms the in vivo studies showing the absence of chloroquine resistance among the positive samples harvested in the six foci of *P. falciparum* malaria transmission. Further studies are in progress to detect multidrug resistance gene *pfmdr1*.

### Sociobehavioural barriers to prevention and treatment of malaria

While various prevention and treatment modalities are available for malaria, a broad range of factors can affect utilization of these options. A review of recent literature suggests barriers at multiple levels including individual, household, socio-cultural, health infrastructure, and policies and programmes.

In relation to utilization of ITNs and long-lasting insecticide-treated nets (LLIN), barriers include: 1) concerns about negative effects of insecticides; 2) feelings of suffocation when using the net; 3) lack of sufficient nets to accommodate number of household members and household sleeping arrangements; 4) perceived lack of privacy due to net construction; 5) over-washing nets resulting in decreased efficacy of the insecticide; and, 6) distrust of the delivery system and associated agencies. At a logistic level, issues for mobile populations include challenges with hanging and dismantling the nets. At the policy and programmes level, barriers include: 1) insufficient stock and product costs; 2) poor programme delivery logistics; 3) a need for coordination of efforts between departments and ministries [37–41].

Barriers to use of intermittent prevention treatment for pregnant women (IPTp) include: 1) limited knowledge of malaria-related risks for mother and child; 2) perceptions of drug safety; 3) poor antenatal care adherence; and, 4) logistical issues related to access to health care, e.g., long lines, distance, and poor healthcare worker-client relationships. Lack of clear healthcare provider guidelines for the use of IPTp also impacts utilization. At the policy and programme levels, IPTp use is limited by insufficient stock and user fees, the need for information about the effectiveness of IPTp, and insufficient funding and slow scale-up of programmes [42–50].

Barriers to diagnosis and treatment of malaria include: 1) use of traditional healers and household treatments; 2) perceptions of personal vulnerability to malaria particularly in low endemic areas; and 3) logistical issues for accessing health care. Among healthcare providers, a lack of familiarity with rapid diagnostic tests and combination therapies can affect diagnosis and treatment. At the policy and programmatic level, limitations include inconsistent RDT supply chains [51–54].

As Haiti moves forward to the elimination of malaria, relevant lessons from these research data include: 1) the need for better understanding of the utility of ITN/LLIN in Haiti in a low-endemic setting; 2) the need for culturally appropriate community-level and health provider training and education in regards to prevention and treatment particularly in relation to vulnerable populations (e.g., pregnant women); 3) the need for long-term education to encourage continued use of prevention strategies, diagnostics and treatment, particularly in low endemic settings; 4) the need for continued programme evaluation and reviews of programme and policy successes; and 5) the need for linkage of preventive strategies across vector-borne diseases (e.g., dengue fever) [55–59].

### Organization of epidemiological surveillance in Haiti

The Ministère de la Santé Publique et de la Population of Haiti created the Direction d’Epidémiologie, de Laboratoire et de la Recherche on November 17, 2005. The mission of the DELR is to guide health action, regulate the norms and procedures of epidemiological activities, coordinate the management of information on priority diseases, and integrate actions of promotion, prevention, and control of diseases.

The DELR conducts surveillance on transmissible and non-transmissible diseases at sentinel sites, which must report certain diseases on either an immediate basis (i.e. cholera, meningitis, rabies, etc.) or a weekly basis (i.e. chikungunya, dengue, malaria, breast cancer, etc.). This mechanism allows the DELR to keep track of: 1) diseases with a somber prognosis; 2) eradicated diseases, or diseases on the road to eradication or elimination); 3) diseases that must be declared at the international level; and, 4) diseases that are of interest for the national health system. To be chosen as a sentinel site for this surveillance, the health facility must see at least 400 patients per month, be accessible, provide a variety of services, have competent, trained and motivated personnel, and have adequate technical capacity. A total of 192 sites cover all geographic areas of Haiti, with an average of one institution covering 56,967 inhabitants.

Each level has its own responsibilities. At the community level, the institution is responsible for identifying cases and notifying the higher levels. At the geographic departmental level, the health team is responsible for analysis, investigation, and reporting. At the national level, the DELR and MSPP are responsible for training, supervision and evaluation. With integrated surveillance at the national level, local response, and the actions of the DELR, Haiti will have a unified system for conducting epidemiological surveillance.

### National control strategy for malaria

A variety of recommendations for strategies can be offered for malaria elimination in Haiti. These are summarized in Table 2. Several national initiatives have been created to specifically address the problem of malaria in Haiti. One initiative, the Service National pour l’Eradication de la Malaria (SNEM—the National Service for Eradication of Malaria) began in 1958 but was closed in 1988. Thus, the Programme National de Contrôle de la Malaria was created in 2005 and permitted the re-launch of anti-malaria activities with funding from The Global Fund. The PNCM is developing a strategic plan for 2016-2022 (Plan Stratégique National d’Elimination de la Malaria—PSNEM) with a vision of being free of malaria throughout the country by 2020. A variety of interrelated goals include coordinating interventions, targeting coastal and low-altitude regions of Haiti, using microscopy or RDTs for all suspected cases, ensuring that all diagnosed cases of malaria in Haiti be due only to imported cases, educating 80 % of the population, and engaging in passive and active surveillance. The 2016-2022 budget is USD $113,412,922.

**Table 2:** Summary of Recommendations for Malaria Elimination Strategies in Haiti

Recommendations for Malaria Elimination Strategies in Haiti
Using Combination Therapy with chloroquine and primaquine to treat suspected and confirmed cases
Using long-lasting insecticidal nets (LLIN) supported by indoor-residual spraying of insecticide
Strengthening infrastructure
Providing malaria diagnosis and treatment
Timely case detection
Complete and accurate case reporting
Expanding and synchronizing surveillance and data systems
Improving field testing for active case detection
Mapping the location of cases and drug resistance
Mapping the location of G6PD deficiency and prevalence
Evaluating testing limitations
Vector control
Controlling malaria in adjacent communities on the border between Haiti and the Dominican Republic
Giving intermittent preventative treatment during pregnancy
Utilization of Community Health Workers
Working with partners to build collaborative, multidisciplinary approaches
Addressing limitations including cost, technical, operational and financial feasibility
Training and public education
Identification of barriers to implementation and use of prevention and treatment modalities from perspectives of multiple stakeholders

### The future: control and research agenda

The malaria control programme in Haiti is preparing to move forward from the pre-elimination control phase to enter into the elimination phase. This will require determining and implementing a novel elimination plan tailored to Haiti. Some of the critical information needed to plan, guide, and sustain malaria elimination in Haiti as described in a 2013 Clinton Health Access Initiative report [2] are these:

- Accurately assess the epidemiology of malaria in Haiti in space and time
- Identify low and high risk areas of transmission in the country, and quantify the population living in each
- Consider both vector control and parasite-based approaches at different levels of coverage and assess the impact of these interventions to reach elimination
- Measure the magnitude of malaria parasite movement on the island and its implications for the elimination effort

A research agenda for Haiti should include improving in vitro culture techniques and testing methods; evaluating drugs to be used for mass drug administration; evaluating new vector control approaches; using new collaborative approaches including modeling to evaluate outcomes of mixed interventions, the role of Community Health Workers, and patient provider attitudes; and strengthening monitoring and evaluation tools that are embedded in the health and social systems.

The efforts of the authors are invested in pre-assessment of these parameters and in capacity building in Haiti to assist in achieving these objectives. Implementation of proven malaria control measures can reduce transmission everywhere in Haiti where malaria occurs. The elimination programme will require continued funding for surveillance and control activities. Recommended activities include collection of data on prevalence to include information from both passive and active surveillance, drug resistance and coverage with malaria interventions. Modelling studies are needed to evaluate persons at risk and technical feasibility to reduce malaria from its baseline to very low levels. More information is needed on coverage gaps for indoor spraying and ITN effectiveness and attitudes around use. Information on vector species present in Haiti, proximity to cases, vector behaviour and resistance to insecticides is needed. Better information from active surveillance and surveys are needed on case detection, diagnosis and treatment and chemoprotection gaps including pregnant women and children is needed. Measures using community health workers and better case management is needed which will reduce the pool of individuals that can affect others and reduce the number of individuals at highest risk of mortality. Moving from control to elimination will require a long timeframe and a sustained commitment.

## Conclusion

Several themes and recommendations emerged during discussions at this scientific meeting. First, more information and research on malaria transmission in Haiti are needed including information from active surveillance of cases and vectors. Second, many healthcare personnel and technicians at clinics throughout Haiti lack adequate training and critical resources on how to properly identify malaria cases so as to improve accurate and timely case reporting. Third, it is necessary to continue studies genotyping strains of *P. falciparum* in different sites with active transmission to evaluate for drug resistance and impacts on health. Fourth, elimination strategies will continue to incorporate use of primaquine in addition to chloroquine and active case surveillance. Elimination of malaria in Haiti will require collaborative multidisciplinary approaches, sound strategic planning, and strong ownership of the Ministère de la Santé Publique et de la Population.

The findings in this report demonstrate that Haiti is continuing to make impressive progress in reducing malaria cases and deaths. Each year, despite formidable challenges, more people are being reached with core malaria interventions, and as a result, more lives are being saved. The last few years saw a major expansion in the use of diagnostic testing, the deployment of combination therapies and improved surveillance systems. Continued progress is needed in vector identification and control. The tremendous achievements are the result of improved and increased commitment, regional initiatives, and continued international and domestic financing. Available funding however remains far less than what is required to protect everyone at risk. Importantly, malaria elimination efforts in Haiti will benefit from a strengthening of health systems. Such investments will help close the disparities in coverage, strengthen disease surveillance and research, and support the development and deployment of new tools and approaches. It will make malaria and other public health responses more effective and more sustainable. Recent progress in reducing the human suffering caused by malaria in many countries has shown us that, with adequate investments and the right combination of strategies, remarkable strides can indeed be made against this foe. Stakeholders should act with a shared and focused goal: to create a Haiti where no one dies of malaria.

## Abbreviations

CDC: Centers for Disease Control and Prevention
DELR: Direction d’Epidémiologie, de Laboratoire et de Recherches [Department of Epidemiology, Laboratory, and Research]
HFHS: Henry Ford Health System
IPTp: Intermittent Prevention Treatment for pregnant women
ITN: Insecticide-Treated Net
LAMP: Loop Mediated Isothermal Amplification
LDR-FM: Ligase Detection Reaction—Fluorescent Microsphere
LLIN: Long-Lasting Insecticide-Treated Net
LNSP: Laboratoire National de Santé Publique [National Public Health Laboratory]
MSPP: Ministère de la Santé Publique et de la Population [Ministry of Public Health and Population]
PCR: Polymerase Chain Reaction
PNCM: Programme National de Contrôle de la Malaria [National Control Plan for Malaria]
PSNEM: Plan Stratégique National d’Elimination de la Malaria [National Strategic Plan for Elimination of Malaria]
RDT: Rapid Diagnostic Test
RFLP: Restriction fragment length polymorphism
SNEM: Service National pour l’Eradication de la Malaria [National Service for Eradication of Malaria]
SNP: Single Nucleotide Polymorphism
